# Chronic Neck Pain Associated with an Old Odontoid Fracture: A Rare Presentation

**DOI:** 10.1155/2013/372723

**Published:** 2013-08-13

**Authors:** Mauricio Avila-Guerra

**Affiliations:** Neuroscience Research Group at Hospital Universitario San Ignacio, Carrera 7 40-62 Hospital Universitario San Ignacio, Second Floor, Bogotá, Colombia

## Abstract

Cervical spine injuries represent a minority of injury cases in motor vehicles accidents but are a real threat to a patient's life. In the wide range of cervical spine injuries, odontoid (dens) fractures represent the most common findings. These fractures are more usually found in the elderly population due to the changes associated with age. Neurological deficit is not frequently found in these injuries. The following is a case presentation of a chronic odontoid fracture with neurological deficit in a young man that was discovered 23 years after he sustained a motor vehicle accident.

## 1. Introduction

It has been reported that the cervical spine is injured in 2.4% of blunt trauma victims [1]. Odontoid fractures account for 9% to 15% of cervical fractures in trauma [2, 3]. More specifically, type II in the Anderson and D'Alonzo classification [4] represents the most common of all odontoid fractures, which are considered unstable fractures. In this type of fracture, the fracture line is at the junction of the odontoid base and the body of the axis (C2 vertebrae) [4].

Neurological deficit is uncommon in patients with odontoid fractures [5]. If the deficit is present, it is most common in male patients that have sustained high velocity injuries and also are at higher risk of dying [6]. 

The scientific literature has reported that the frequency of missed injuries in the cervical spine varies from 4% to 30% [7, 8].

This paper presents a case of a patient with a chronic odontoid fracture who developed a pseudarthrosis (pannus) of bone during fracture healing that sustained a high-velocity injury in a motorcycle accident 23 years before presenting to the emergency department. The case reported here mixes uncommon conditions in odontoid fracture: a missed fracture of the odontoid process, neurological deficits, and the 23 years span between the fracture and the deficit.

## 2. Case Presentation

A 37-year-old male patient presented to the emergency department complaining of loss of strength in his upper right arm, right hand, and paresthesias along the arm. He also complained of neck pain that has been going on and off for about 2 years. The neurological symptoms appeared three months prior to the consultation. General examination was unremarkable. The initial neurological exam showed an upper right limb paresis, and the rest of the exam was normal. The patient had no recent travels, and lived with his wife and a son. The patient referred had no recent trauma to the head or the neck or any type of vehicle accident.

The initial clinical suspicion was a spinal cord compression at the upper cervical spinal cord, primarily due to the neck pain and the upper limb paresis that locate the lesion in this particular area.

**Figure 1:**
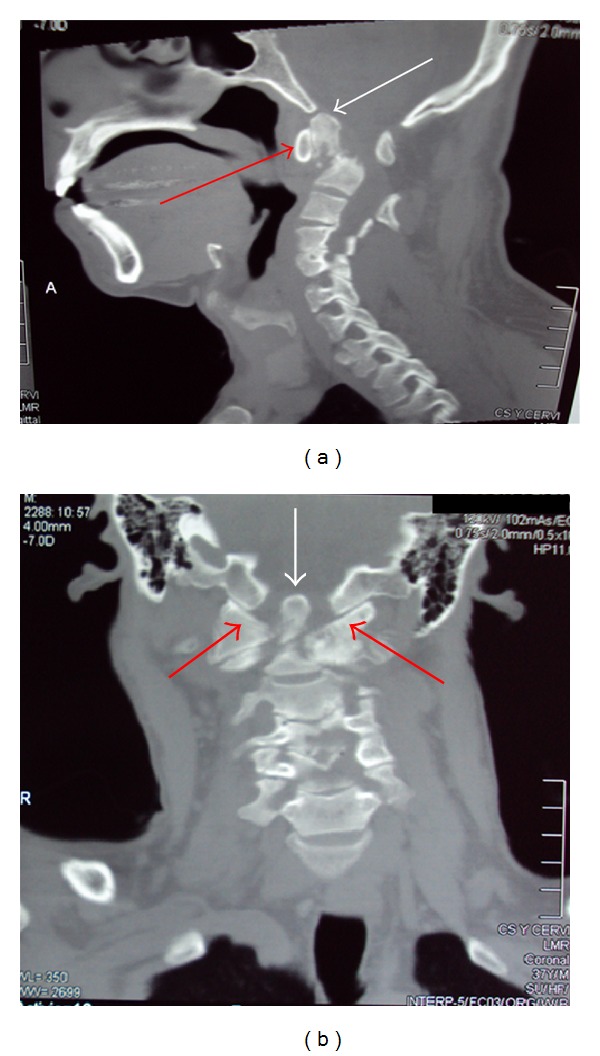
Initial CT of the cervical spine. (a) Sagittal image. Red arrow indicates the anterior arch of the C1 vertebrae (Atlas). The white arrow indicates the odontoid process fractured from the rest of the C2 vertebrae. (b) Coronal image of the initial CT. White arrow: odontoid process of C2 vertebrae with the fracture seen underneath it. Red arrow: lateral masses of the Atlas (C1 vertebrae).

A noncontrast Computed Tomography (CT) was obtained to evaluate spine integrity (Figure 1). A mass at the level of the odontoid process was seen with density similar to bone that corresponded to an old fracture of the odontoid process. Due to the changes noted in the density of the bone, the fracture has healed as pseudarthrosis. An important cervical canal stenosis was noted and immediately the patient was put in a rigid cervical collar to prevent any further damages to the spinal cord.

**Figure 2:**
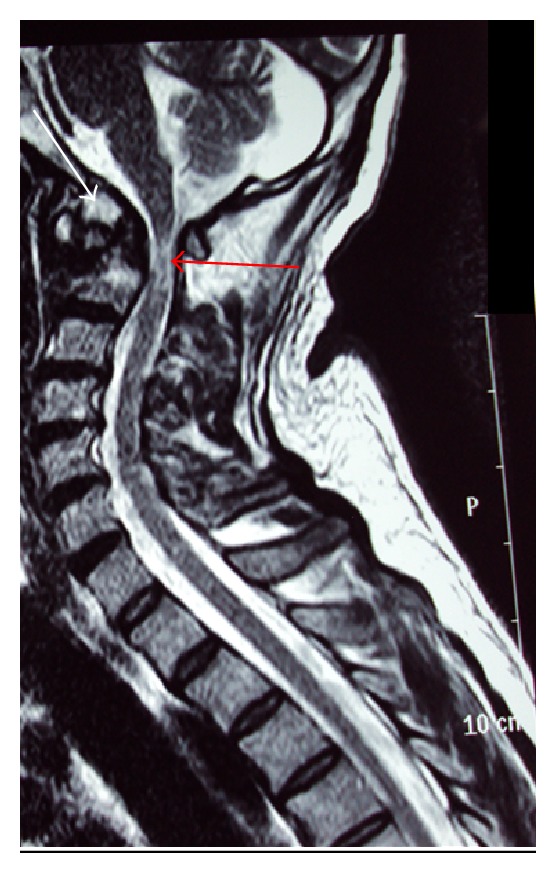
MRI in T2 sequence of the cervical spine. Red arrow: chronic myelopathy (hyperintense signal) can be seen. Anterior to that the bone mass (pseudarthrosis) is seen. The white arrow is indicating the odontoid process of the axis (C2 vertebrae).

Due to the high risk of myelopathy, Magnetic Resonance Image (MRI) of the cervical spine was ordered (Figure 2). Signs of myelopathy are seen behind the mass of bone that was detected in the CT at the C1-C2 spinal cord levels, confirming the images on the CT that the fracture was in fact an old one, and the clinical signs of the patient were due to this injury in the spinal cord. 

On a thoroughly second interrogation, the patient revealed a motorcycle accident that happened 23 years before this visit and listed a scalp laceration as the sole injury he had sustained at that time.

The patient was admitted for immediate surgery for decompression of the spinal canal. Transoral resection of odontoid process was scheduled. At the same time of the surgery an occipitocervical arthrodesis for cervical spine stabilization was also prepared. The surgery was performed without complications and the patient was then moved to the surgical Intensive Care Unit (ICU) for surgical follow-up. A CT was ordered after the surgery for a follow-up on the spine injuries. Adequate positions of the screws were seen, and almost complete removal of the pseudarthrosis was achieved.

After four days in the intensive care unit, the patient continued his recovery at the neurosurgical hospitalization floor.

At day five of surgery, the patient presented with an acute respiratory failure secondary to obstruction of the upper airway and needed respiratory resuscitation and an emergency tracheostomy due to the difficult airway (secondary to the occipito-cervical arthrodesis, a hyperextension of the neck was difficult, and the high risk of spinal cord damage was primarily because of the short time between the surgeries). The patient was moved back to the ICU and continued his treatment with an orogastric feeding tube for 13 days waiting for the resolution of pharyngeal edema.

Twenty-five days after the surgery, the patient was discharged to his home with the tracheostomy and without any need of gastric feeding tube. The paresthesias were in process of resolution and the paresis was slightly improving.

The follow-up consult was conducted one week after the surgery, neurological examination persists with a slight paresis on his right arm, he regained strength in the hand, and the paresthesias disappeared. The patient continued respiratory therapy for the tracheostomy management and was able to remove the tracheostomy tube 3 months after surgery.

## 3. Discussion

As it was shown before, odontoid fractures are common findings in cervical spine trauma. However, neurological deficits are not common when patients have an odontoid fracture [5]. We performed a literature review in MEDLINE database through PubMed to find articles similar to our case presentation. MeSH terms were used in different search orders to try to maximize the database search. There was no limit of time for the publication of the paper nor language limits. The search strategy is the following ((“Odontoid Process/abnormalities”[MeSH] OR “Odontoid Process/injuries”[MeSH] OR “Odontoid Process/physiology”[MeSH] OR “Odontoid Process/physiopathology”[MeSH] OR “Odontoid Process/radiography”[MeSH] OR “Odontoid Process/surgery”[MeSH]) AND “Spinal Fractures”[MeSH]) AND “Chronic Disease”[MeSH]. The results showed 3 articles that fit the search criteria; two were excluded due to the language [9, 10] (German in both cases) and the other one was reviewed. The study by Blacksin and Avagliano [11] presented 3 cases of chronic odontoid fracture. The maximum time span between the injury and the diagnosis was 1 year in the three patients examined. The diagnostic criteria used by the authors to determine the chronicity of a fracture were also used to the images of the patient presented here, and all of them fit in the category of chronic fracture of the odontoid process, confirming the initial diagnosis in the CT of the patient presented here.

Since the publication of the Anderson and D'Alonzo classification for odontoid fractures [4], multiple studies have shown that the type II fractures have the highest incidence of nonunion in the bone. Different rates have been reported ranging from 12 to 63% of nonunion in these types of fractures [2, 12, 13]. 

The surgical management was only possible by the transoral route [14] in our patient, primarily because the abundant bone pseudarthrosis was found in preoperative imaging, and there is a possibility of a full resection of odontoid process and anterior arch of Atlas (C1 vertebrae) via this surgical entry point. Occipito-cervical arthrodesis was mandatory for spine stabilization after the initial procedure and was performed in the same surgical day.

In the case presented here, it is highly probable that the patient presented with an odontoid fracture at the motorcycle accident that he suffered 23 years before presentation. We confirmed that the fracture was not an acute one at presentation due to the odontoid fragment characteristics in the CT as described by Blacksin and Avagliano [11]. Neurological deficits are uncommon with this type of fractures [5, 15], combined with the high percentage of missed fractures and probably the low resolution of the equipment or the unavailable use of CT at the time of the fracture 23 years ago, all contributed to miss the fracture at that given time. The long years that the fracture sustained continuous movements of the neck, and the unawareness of the patient of his condition probably contributed to the formation of the mal-union of the fracture, the abnormal regrowth of the bone, and the formation of pseudarthrosis.

It was not until the pseudarthrosis damaged the spinal cord causing myelopathy that the patient started to have some neurological deficits that prompted the emergency visit.

To the best of our knowledge, this is the first case of cervical myelopathy that developed 23 years after a fracture was reported in the literature.

Emergency department physician must be careful when evaluating patients with neurological deficit in the upper extremities due to the high probability of finding a spinal cord injury in the cervical spine. The case presented here illustrates how every patient with neck pain must be evaluated thoroughly before proceeding to any other type of tests or even physical mobilization of the neck.

Any neurological deficit in the upper extremities with any history of old trauma, even if the patient considered it minor, should be addressed as a probable spine cord injury at the level of cervical spine.

Although uncommon in the young adults, odontoid fractures must also be taken into consideration when evaluating a patient that sustained a motor vehicle accident.

Finally, time should not be a bias to rule out any type of cervical spine fractures as it was demonstrated in this case.
